# Efficacy of Quality Criteria to Identify Potentially Harmful Information: A Cross-sectional Survey of Complementary and Alternative Medicine Web Sites

**DOI:** 10.2196/jmir.6.2.e21

**Published:** 2004-06-29

**Authors:** Muhammad Walji, Smitha Sagaram, Deepak Sagaram, Funda Meric-Bernstam, Craig Johnson, Nadeem Q Mirza, Elmer V Bernstam

**Affiliations:** ^1^University of Texas Health Science Center at HoustonSchool of Health Information SciencesHouston TXUSA; ^2^Department of Surgical OncologyThe University of Texas M.D. Anderson Cancer CenterHouston TXUSA

**Keywords:** Quality, harm, Internet, medical information, World Wide Web, complementary and alternative medicine

## Abstract

**Background:**

Many users search the Internet for answers to health questions. Complementary and alternative medicine (CAM) is a particularly common search topic. Because many CAM therapies do not require a clinician's prescription, false or misleading CAM information may be more dangerous than information about traditional therapies. Many quality criteria have been suggested to filter out potentially harmful online health information. However, assessing the accuracy of CAM information is uniquely challenging since CAM is generally not supported by conventional literature.

**Objective:**

The purpose of this study is to determine whether domain-independent technical quality criteria can identify potentially harmful online CAM content.

**Methods:**

We analyzed 150 Web sites retrieved from a search for the three most popular herbs: ginseng, ginkgo and St. John's wort and their purported uses on the ten most commonly used search engines. The presence of technical quality criteria as well as potentially harmful statements (commissions) and vital information that should have been mentioned (omissions) was recorded.

**Results:**

Thirty-eight sites (25%) contained statements that could lead to direct physical harm if acted upon. One hundred forty five sites (97%) had omitted information. We found no relationship between technical quality criteria and potentially harmful information.

**Conclusions:**

Current technical quality criteria do not identify potentially harmful CAM information online. Consumers should be warned to use other means of validation or to trust only known sites. Quality criteria that consider the uniqueness of CAM must be developed and validated.

## Introduction

Online health information can harm as well as heal. Many quality criteria have been suggested to help consumers identify misleading, inaccurate, or harmful information. Objective quality criteria that offer a limited number of options are particularly promising since they are easier to assess. For example, it is easier to assess whether an author is identified than to determine whether the author is qualified. However, even seemingly objective quality criteria have proven unreliable without specific operational definitions [1]. Further, there is little evidence that these criteria, known as "technical criteria," actually filter out undesirable health information. The few studies that have attempted to evaluate technical criteria reported conflicting results [2-4]. If harmful information can be effectively identified, this should be publicized. If, on the other hand, currently available quality criteria cannot identify potentially harmful information, then we should caution consumers and work on finding other ways of identifying problematic information online.

In this study, we analyze Web sites that display information about complementary and alternative medicine (CAM). CAM includes "diverse medical and healthcare systems, practices and products that are not presently considered to be a part of conventional medicine," such as dietary supplements, aromatherapy, chiropractic, and homeopathy [5]. Assessing accuracy and quality of CAM Web sites poses unique challenges as there is less documented research on the efficacy of CAM products, yet use is common and the potential for harm remains. There is also no gatekeeper to control and monitor access to CAM. Consumers can choose the product and dosage without having to encounter a healthcare professional. In fact, patients often fail to report CAM use to their physicians [6]. On the other hand, consumers frequently turn to the Internet to answer questions about CAM, and trust and act upon what they see online [7]. However, CAM information online has been found to be commercially driven [8], to be poorly referenced [8], and to contain illegal claims [9], and it may therefore be dangerous to consumers [10]. The combination of accessible, unproven CAM therapies and poor quality online CAM information is dangerous.

"Accuracy is a function of whether a site reflects the use of … agreed-upon benchmark[s] such as clinical practice guidelines." [11] The accuracy of CAM information, which is often not evidence-based and lacks support from the peer-reviewed biomedical literature, is not testable. However, we can assess the potential harm of displayed information, even if we cannot verify its accuracy. Further, if information regarding the safety and efficacy of a product is available, it should be displayed.

Our previous work provides preliminary evidence that breast cancer Web sites that meet more technical quality criteria are less likely to contain false statements [12]. Motivated by a desire to help consumers, we sought to determine whether current technical quality criteria can identify potentially harmful CAM information.

## Materials and Methods

### Selection of Web Sites

Consumers use general-purpose search engines rather than medical sites or portals to find information, and most do not go beyond the first page of search results [13]. Therefore, we chose the ten most popular search engines (Table 1) to select Web sites that consumers are likely to encounter [14]. The three most popular herbs in the United States (in terms of dollars spent) [15], ginseng, ginkgo, and St. Johns wort, and their most common uses formed the search query. The following three queries were executed in each search engine on July 15, 2003: "ginseng and cancer," "ginkgo and memory loss," and "St. John's wort and depression." All Web sites listed on the first results page, including sponsored or paid links, were analyzed.

**Table 1:** Search engines used to select Web sites

Search Engine
1. Google
2. Yahoo
3. MSN
4. AOL
5. Ask Jeeves
6. Overture
7. Infospace
8. Netscape
9. AltaVista
10. Lycos

A Web site was included if it contained at least one sentence or phrase of health information on the search topic. Health information was defined as "information intended to be used to maintain or improve health, including to understand disease processes, health care issues, etc… to prevent, diagnose, or treat health problems, to be rehabilitated from the effect of diseases, or treatments, and to seek and select health care plans, providers, and other resources." [16] Duplicate URLs were removed. HTTrack [17], a Web site copier was used to permanently capture each Web site and every directly linked page.

### Assessing Technical Quality Criteria

In prior work, we assessed inter-rater agreement for popular technical quality criteria [1]. We assessed the degree to which two raters agreed upon the presence or absence of 22 quality criteria selected from Eysenbach's systematic review [17] of a sample of 21 CAM Web sites. Our preliminary analysis showed poor inter-rater agreement on 10 of the 22 criteria. Therefore, we created operational definitions for each of the criteria, decreased the allowed choices, and defined a location to look for the information. As a result, 15 out of the 22 quality criteria had acceptable inter-rater agreement (kappa > 0.6).

For this study, one evaluator (MW) analyzed all Web sites for compliance with 15 technical quality criteria (Table 2) that we previously determined to be reliably assessable. Therefore, in this study we did not re-calculate inter-observer reliability for these technical criteria.

### Assessing Potential Harm

First, a set of critical facts for each of the three herbs was determined by consensus of two clinically trained reviewers (SS, DS); please see appendices 1-3. The sets of critical facts were extracted from two independent sources of CAM information: the Physician Desk Reference (PDR) for Herbal Medicines [19] and the Sloan Kettering database of herbs [20]. After the sets of critical facts were determined, the CAM content displayed on each Web site was independently evaluated by both reviewers. Cases where reviewers disagreed were resolved by consensus. In order to minimize bias, materials identifying each Web site's origin, such as organization name, logo, footers, URLs and hyperlinks were removed. However, no changes were made to the design or layout.

In order to verify the concordance between reviewers, two additional clinically trained evaluators (validation reviewers), who were not aware of the study hypothesis or quality criteria tested, were given 30 randomly selected sites from the same sample looked at by primary reviewers (SS, DS). Inter-rater agreement between the validation reviewers was calculated. The validation reviewers were given the same critical facts documents as the primary reviewers and each validation reviewer assessed every site independently. After each reviewer independently evaluated the Web sites, inter-rater agreement was calculated between the two validation reviewers. Subsequently, cases of disagreement were resolved by consensus. A second inter-observer agreement measure was calculated between the pairs of reviewers (primary reviewers vs. validation reviewers) based on the consensus data.

Content on each page was scrutinized for the presence of misleading statements likely to cause physical harm (acts of commission) and for vital information that was missing (acts of omission). Commission may be thought of as a surrogate for accuracy, while omission has been referred to as completeness, coverage, or comprehensiveness [21]. We based our evaluation on the following framework, adapted from Markman [22]:

1.
  - Direct toxicity
  - Interaction with conventional medical therapy
  - Delay in diagnosis or conventional treatment
  - Avoidance of conventional treatment
2.
  - Warnings
  - Drug interactions
  - Contraindications
  - Side effects

Statements that suggest use of higher doses of herbs than recommended in the critical facts documents (appendices 1-3) were categorized as causing "direct toxicity." Statements suggesting that the herb protects against disease and encouraging patients to self-medicate instead of seeing a physician were placed in the "delay in diagnosis or conventional treatment" category. Statements that project herbs as an "alternative to conventional treatment" (for example, "the herb is the first choice of treatment for the disease") were categorized as potentially causing "avoidance of conventional treatment." Statements that suggested using herbs with medications known to have drug interactions (for example, using St. John's wort with monoamine oxidase inhibitors) were classified as causing potential harm due to "interaction with conventional therapy." However, while evaluating potential physical harm due to omission of information about interactions, we did not expect Web sites to list all the drug interactions listed in the critical facts documents. Web sites that noted at least one drug interaction were considered not to omit drug interaction information. Web sites with vague statements such as "there are many interactions," were categorized as having "omitted drug interactions." Potential physical harm was present if any error of commission or omission was found.

We recognize that in addition to physical harm due to either commission or omission, CAM information on the Internet may cause other types of harm, such as emotional and financial. Emotional harm may occur because of inaccurate perception of disease or conventional therapy such as exaggeration of side effects of conventional treatment and presentation of alternative treatment as a "natural cure." Financial harm may be caused by the purchase of ineffective or harmful yet expensive CAM products. However, we did not evaluate emotional and financial harm in this study because of the inherent subjectivity involved, and difficulty in quantifying and assessing such measures.

### Statistical Analyses

The dichotomous (yes/no) dependent variables were: 1) physical harm from commission and 2) physical harm from omission. The independent variables were also dichotomous and consisted of the 15 technical quality criteria listed in Table 2. In addition, these 15 criteria were grouped into 5 categories [23]: authority, transparency and honesty, updating of information, editorial policy, and other. Web sites were classified into two groups based on whether they complied with the median number of quality criteria. The first group complied with six or fewer technical quality criteria, the second group complied with more than six technical quality criteria.

Inter-observer agreement measures were calculated to assess a) the degree to which validation reviewers agreed among themselves in their assessments of these dichotomous dependent variables (Table 3) and, b) the degree to which the validation reviewers agreed with the primary reviewers (Table 4). Cohen's kappa (K) is a commonly used measure of inter-observer agreement between two observers for dichotomous data. However, because K is affected in complex ways by the presence of bias between observers and by the distributions of data across the categories [24], we computed the prevalence-adjusted bias-adjusted kappa (PABAK), the bias index (BI) and the prevalence index (PI), as well as K, as recommended by Byrt et al [24].

The bias index (BI) is defined as the difference between the proportions of "Yes" for the two raters. The prevalence index (PI) is defined as the difference between the probability of "Yes" and the probability of "No." A BI close to 0 indicates less bias, while values closer to 1 (absolute value) indicate greater bias. Similarly, a PI close to 1 (absolute value) indicates high prevalence, while a PI closer to 0 indicates lower prevalence. The BI then measures the degree to which one reviewer tends to identify more or fewer occurrences than the other, while the PI measures the degree to which "Yes" agreements or "No" agreements predominate. The PABAK index of agreement between two observers is a measure that adjusts for both bias and prevalence. Although the derivation of the PABAK index is somewhat more complex, in practice it can be calculated as 2P_0_ - 1, where P_0_ is the proportion of observed agreement. Consequently, PABAK ranges from -1 to +1 and like K, a value of 0 represents no better than chance agreement, while magnitudes approaching 1 indicate maximal agreement.

Chi-square was calculated for each pairing of an independent variable with a dependent variable. Given the large number of statistical tests performed, significance was set at α*<*0.01. All analyses were performed using SPSS 11.0 statistical software.

## Results

A total of 546 Web sites were retrieved. After removing duplicates and checking for eligibility, 150 Web sites remained: 54 for the query "ginseng and cancer," 46 for "ginkgo and memory loss," and 50 for "St. John's wort and depression."

**Table 2:** Compliance of CAM Web sites with technical quality criteria. Criteria are also grouped into 5 categories (in bold). Values are counts (percentages)

**Quality criteria**	**Number of Web sites (%)**
**Authority**	
Disclosure of authorship	41 (27)
Author's credentials disclosed	17 (11)
Credentials of physicians disclosed	2 (1)
Author's affiliation disclosed	17 (11)
**Transparency and Honesty**	
Sources clear	100 (67)
General disclosures	147 (98)
References provided	54 (36)
Disclosure of ownership	144 (96)
**Currency/ Updating of information**	
Date of creation disclosed	31 (21)
Date of last update disclosed	21 (14)
Date of creation or update disclosed	49 (33)
**Editorial Policy**	
Editorial review process	9 (6)
**Others**	
Internal search engine present	78 (52)
Feedback mechanism	132 (88)
Copyright notice	105 (70)

### Technical Quality Criteria

Most Web sites did not comply with technical quality criteria. On average, a Web site complied with 6.3 (SD±2.6) of 15 criteria. One site failed to comply with any criteria, while three sites complied with 13 criteria. Only 27% of sites disclosed authorship, 36% provided references and 6% mentioned an editorial review process. Table 2 shows the number of Web sites that complied with each of the 15 quality criteria.

### Assessing Potential Harm: Agreement among Reviewers

As shown in Table 3, agreement between the two evaluation reviewers was high (all PABAK > 0.67). Although there was little bias, there was a strong prevalence effect. Therefore, the two validation reviewers had a high degree of agreement for all measures of harm from commission and omission. Similarly, as shown in Table 4, consensus agreement between the primary and validation reviewers was also high (all PABAK > 0.73).

**Table 3:** Agreement among validation reviewers on a sample of 30 Web sites

	**P_0_**	**BI**	**PI**	**K**	**PABAK**
A. Physical Harm-Commission*	0.87	0	-0.8	0.259	0.73
Direct Toxicity	0.93	0.07	0.93	Undefined	0.87
Interactions	0.97	-0.03	0.97	Undefined	0.93
Delay in diagnosis	1	0	-1	Undefined	1
Avoidance of conventional therapy	0.97	-0.03	-0.9	0.651	0.93
B. Physical Harm-Omission*	0.97	0.03	0.97	Undefined	0.93
Omission of Warnings	0.93	0.07	0.8	0.634	0.87
Omission of Drug Interactions	0.97	-0.03	0.7	0.87	0.93
Omission of Contraindications	1	0	0.8	1	1
Omission of Adverse Reactions	0.83	-0.17	0.77	0.242	0.67

^*^ P_0_= observed agreement,BI = bias index, PI = prevalence index, K = Cohen's kappa, PABAK = prevalence-adjusted bias-adjusted kappa. Undefined = SPSS did not compute value due to zero variability in a variable.

**Table 4:** Agreement between primary and validation reviewers on a sample of 30 Web sites

	**P_0_**	**BI**	**PI**	**K**	**PABAK**
A. Physical Harm-Commission*	0.93	0.07	-0.73	0.71	0.87
Direct Toxicity	0.93	0.07	-0.8	0.63	0.87
Interactions	1	0	-1	Undefined	1
Delay in diagnosis	0.93	0.07	-0.93	Undefined	0.87
Avoidance of conventional therapy	1	0	-0.93	1	1
B. Physical Harm-Omission*	0.97	0.03	0.97	Undefined	0.93
Omission of Warnings	0.9	0.03	0.83	0.35	0.8
Omission of Drug Interactions	0.97	-0.03	0.7	0.87	0.93
Omission of Contraindications	0.97	-0.03	0.77	0.84	0.93
Omission of Adverse Reactions	0.87	0.07	0.73	0.43	0.73

^*^ P_0_= observed agreement, BI = bias index, PI = prevalence index, K = Cohen's kappa, PABAK = prevalence-adjusted bias-adjusted kappa. Undefined = SPSS did not compute value due to zero variability in a variable.

**Table 5:** Number of CAM Web sites that display potentially harmful information. Values are counts (percentages)

**Type of Harm**	**Number of Web sites (%)**
A. Physical Harm-Commission*	38 (25)
Direct Toxicity	19 (13)
Interactions	12 (8)
Delay in diagnosis	5 (3)
Avoidance of conventional therapy	10 (7)
B. Physical Harm-Omission*	145 (97)
Omission of Warnings	121 (81)
Omission of Drug Interactions	124 (83)
Omission of Contraindications	134 (89)
Omission of Adverse Reactions	125 (83)

^*^ Note: Totals in these rows are calculated if any of the four categories of commission or omission were found on the Web site.

### Potential Harm

Potential physical harm from omission was more prevalent than from commission (97% vs. 25%, Table 5). However, a substantial number of Web sites (25%) displayed statements that could lead to physical harm. Statements that may cause toxicity if acted upon (direct toxicity) were present in 13% of CAM Web sites, while 7% of Web sites included statements encouraging the avoidance of conventional therapies. Eight percent of sites included information that may lead to harm from interactions if the advice were followed. Most CAM Web sites (97%) omitted vital information such as contraindications (89%) and drug interactions (83%).

### Technical Quality Criteria

We found that individual technical quality criteria did not identify sites with the potential to cause physical harm from commission or omission (Table 6). Similarly, when technical criteria were grouped into categories (such as authority, transparency and honesty, etc.), no significant association was found with potential physical harm (Table 7). Even when Web sites were classified into two groups, those complying with more criteria (≥ 6) versus fewer criteria (<6), there was no significant relationship. Overall, 44 hypotheses were tested but none were significant at the α<0.01 level, despite our study having 0.80 power to detect significance. Surprisingly, the presence of two quality criteria where a significant association was found at α<0.05 ("sources clear" and "editorial review process") indicated a greater chance of potential harm; the reverse of their original intent. However, it is possible that these two significant results may be due to chance since we conducted numerous statistical analyses.

**Table 6:** Association between individual quality criteria and potential harm. Values are counts (percentages of Web sites complying with that criterion)

	Total number of Web sites complying with criterion	Physical harm by					
		Commission			Omission		
		Present (n = 38)					
Disclosure of authorship	41	11 (29)	30 (27)	0.80	39 (27)	2 (40)	0.52
Author's credentials disclosed	17	3 (8)	14 (12)	0.44	16 (11)	1 (20)	0.53
Credentials of physicians disclosed	2	1 (3)	1 (1)	0.42	2 (1)	0 (0)	0.79
Author's affiliation disclosed	17	6 (16)	11 (10)	0.32	17 (12)	0 (0)	0.42
Sources clear	100	31 (82)	69 (62)	0.02	95 (65)	5 (100)	0.11
Date of creation disclosed	31	8 (21)	23 (20)	0.95	30 (21)	1 (20)	0.97
Date of last update disclosed	21	5 (13)	16 (14)	0.86	20 (14)	1 (20)	0.69
Date of creation or update disclosed	49	13 (34)	36 (32)	0.81	47 (32)	2 (40)	0.72
General disclosures	147	37 (97)	110 (98)	0.75	142 (98)	5 (100)	0.75
References provided	54	14 (37)	40 (36)	0.9	51 (35)	3 (60)	0.26
Disclosure of ownership	144	35 (92)	109 (97)	0.16	139 (96)	5 (100)	0.64
Internal search engine present	78	21 (55)	57 (51)	0.64	75 (52)	3 (60)	0.72
Feedback mechanism	132	32 (84)	100 (89)	0.41	128 (88)	4 (80)	0.58
Copyright notice	105	31 (82)	74 (66)	0.07	100 (69)	5 (100)	0.13
Editorial review process	9	5 (13)	4 (4)	0.03	9 (6)	0 (0)	0.57

**Table 7:** Association between groups of technical quality criteria and potential harm. Values are counts (percentages of Web sites complying with that criterion)

	Total number of Web sites complying with criterion	Physical harm by					
		Commission			Omission		
		Present (n = 38)					
Authority	41	11 (29)	30 (27)	0.80	39 (27)	2 (40)	0.52
Transparency and honesty	149	37 (97)	112 (100)	0.09	144 (99)	5 (100)	0.85
Currency/updating of information	51	13 (34)	38 (34)	0.98	49 (34)	2 (40)	0.77
Editorial policy	9	5 (13)	4 (4)	0.03	9 (6)	0 (0)	0.57
Others	139	34 (90)	105 (94)	0.38	134 (92)	5 (100)	0.52

### Top Level Domain

We also explored the relationship between top level domain and potential harm. Seventy-seven percent of the 150 Web sites analyzed were commercial (.com), 10% organizational (.org), 7% network (.net), 3% educational (.edu), 2% governmental (.gov) and 1% unknown (numerical IP address only). Fisher's exact test statistic was calculated as expected values in some cases were <5, and significance was set at α = 0.05 level. Only the network top level domain had a significant relationship with physical harm from omission (Table 8). Of the 10 Web sites with the network top level domain, 20% did not contain harm from omission. In contrast, only 2% of sites that had a top level domain other than network did not have harm from omission (p<0.04). However, there was no statistically significant relationship between network and non-network sites with respect to physical harm from commission. Although there were few educational and government sites in our study, it is notable that there were no identified cases of potential harm by commission in these sites. As most Web sites were commercial, it is difficult to draw meaningful conclusions from this analysis.

**Table 8:** Association between top level domain and potential harm. Values are counts (percentages of Web sites complying with that top level domain)

	Total number of Web sites with top level domain	Physical harm by					
		Commission			Omission		
		Present (n = 38)					
	116	31 (82)	85 (76)	0.65	114 (79)	2 (40)	0.07
	10	3 (8)	7 (6)	0.71	8 (6)	2 (40)	0.04*
	4	0 (0)	4 (4)	0.57	4 (3)	0 (0)	1.0
	16	4 (11)	12 (11)	1.0	15 (10)	1 (20)	0.43
	3	0 (0)	3 (3)	0.57	3 (2)	0 (0)	1.0
	1	0 (0)	1 (1)	1.0	1 (1)	0 (0)	1.0

^*^ Note: Fisher's exact test calculated as expected values in some cases were <5

### Intent to Sell Products

In order to explore the relationships between Web sites that sold products and those that did not, two evaluators independently revisited each Web site and identified Web sites that allowed the ordering of products. Agreement between reviewers was high (K=0.95). Fifty-three percent of Web sites (n=79) sold products. There was no significant relationship between selling products and potential harm due to omission (P=0.56) or commission (P=0.02). Although not statistically significant at the α = 0.01 level, selling products was actually related to less harm from commission, the reverse of what we would expect. In fact 63% (n=24) of the harmful Web sites from commission were found on sites that did not sell products, while 37% (n=14) were found on Web sites that sold products. Therefore, in our sample there does not appear to be more harmful information on sites that sell products.

## Discussion

We found that most CAM Web sites were potentially harmful either by displaying statements which could cause harm, or by omitting vital information. However, our data suggest that available technical quality criteria fail to identify potentially harmful information online.

We found that one quarter of CAM Web sites present information that may cause physical harm if acted upon. These sites encouraged consumers to avoid conventional therapy, presented information on products that may be directly toxic, or presented information on products that may cause interactions with conventional medications. This is potentially dangerous because consumers have easy access to CAM products online and act upon what they see on the Internet [7], often do so without the knowledge or advice of clinicians [25].

Almost all (97%) CAM Web sites omitted vital warnings, drug interactions, contraindications, or adverse reactions. This is concerning because many consumers perceive "natural" products as safe. Further, many herbs that may be safe when used alone interact with conventional medications.

Previous studies have found scientific references [4], absence of financial interest [4], display of copyright [2], and display of editorial policy [3] to correlate with information accuracy. Technical quality criteria evaluated in this study may be unsuitable for CAM information as they seek to identify accuracy, which is difficult to determine for CAM. Surprisingly, even generally accepted measures of content quality such as disclosure of authorship and updating of information had no relationship to potential harm. Other researchers have also encountered difficulty in developing guidelines to evaluate CAM information [26].

Our previous study of breast cancer information online found that sites which complied with >3 JAMA benchmarks [27] (authorship, references, currency, and disclosure) were more accurate than lower quality sites (<3 JAMA benchmarks) [12]. However, in this sample of CAM Web sites we found no such relationship for potential harm resulting from commission (p=0.31) or omission (p=0.21). We are forced to question the assumption, at least for CAM information, that consumers can be taught to discern good content from bad by looking at domain-independent quality criteria. Recommending such criteria may convey a false sense of security, inadvertently causing consumers to trust harmful CAM websites. Although the technical criteria we assessed had no relationship to potential harm, other criteria or tools not tested may have some value.

**Table 9:** Web sites that contained no errors (neither commission nor omission)

Company/Organization	Selling Products	Top Level Domain
American Cancer Society	No	.org
About Inc	No	.com
Pagewise Inc	No	.com
Natural Pharmacy	Yes	.net
Vitamin Trader	Yes	.com

Five Web sites contained no harmful information from either commission or omission at the time of our study (Table 9). Four of the five best performing Web sites were retrieved from a search for St. John's wort, and one from a search on ginseng. One of these Web sites was from the American Cancer Society. However, the remaining four Web sites were from commercial or for-profit entities, two of which sold products. We note that Web site content changes frequently. Therefore, it is difficult to endorse any list of Web sites.

The major limitation of our study is the inherently subjective domain. Whether or not information has the potential to harm a consumer is a subjective clinical judgment which defies strict definition. However, relatively high inter-observer agreement among clinically trained reviewers suggests that our definitions were consistent.

Our study was also limited by our sample, which was restricted to Web sites displaying information about three popular herbs. Searches on other herbs or different alternative therapies may have different results. Also, we did not evaluate all possible technical quality criteria. Instead, we evaluated only criteria that were used in three or more studies as reviewed by Eysenbach et al [18] and were found to be reliably assessable using pre-determined operational definitions [1]. It is possible that other quality criteria will be more effective.

Since the primary reviewers (SS, DS) were aware of the study hypotheses, they may have been biased by this knowledge. However, inter-observer agreement between the primary and validation reviewers (who were unaware of the hypotheses) was high. Therefore this potential bias appears to have minimal effect on the results.

As we search for quality measures, we must keep in mind that some potentially useful criteria are easily manipulated. For example, one study found sites that claimed copyright were more accurate [2]. Such very specific and objective criteria are appealing since they may be automatically assessed using software, and evaluated by consumers by simply searching for the word "copyright" or © symbol. However, it is easy for site builders to claim copyright without changing the health information displayed on their site.

Although we restricted our analysis to individual sites, consumers may not make health-care decisions on the advice of one site, but rather on the collective information learned, confirmed or refuted from a multitude of online sources. Future work can assess the degree to which confirmatory evidence present on a range of sites can screen out undesirable information. In addition, it would also be important to understand why consumers search for CAM information. After all, some may turn to CAM only after conventional treatment fails, whereas others may reject traditional therapies.

The Internet provides a constantly changing, endless variety of information from innumerable sources. Ideally, we would like to empower consumers to evaluate health information for themselves. Currently available technical quality criteria, however, are not adequate to evaluate CAM information. For the time being, it may be prudent to recommend that consumers looking for CAM information online rely on known, authoritative providers of information. With this in mind, we must continue to search for ways of alerting consumers to potentially harmful information without restricting them to known sources.

## Appendix 1

### Critical Facts: St. John's Wort *(hypericum perforatum)*

INTRODUCTION

Also known as Saint Johns wort, hypericum, goatweed, God's wonder plant, witches herb. Generally is used for depression, seasonal affective disorder, and anxiety. St. John's wort should not be used for patients with severe depression. Studies also show possible efficacy in the management of anxiety and premenstrual syndrome, although additional research is necessary.

INDICATIONS AND USAGE

- Anxiety, depression, fatigue, insomnia, pain, pediatric nocturnal incontinence, premenstrual syndrome, seasonal affective disorder (SAD), depressive moods, inflammation of the skin, blunt injuries, wounds and burns.

WARNINGS

- May cause photosensitivity.
- St John's wort should be discontinued one week before surgery or chemotherapy.

CONTRAINDICATIONS

- Pregnant or nursing women should not consume.
- Simultaneous use of a MAO inhibitor-St. John's wort contains some weak MAOI properties that may add to the effects of other MAOI drugs therefore increasing the risk for hypertensive crisis.

ADVERSE REACTIONS

- General: No health hazards are known in conjunction with the proper administration of designated therapeutic dosages. Tannin content may lead to digestive complaints, such as feeling of fullness or constipation. Patients with previous history of photosensitization to various chemicals should be cautious of direct sun exposure.
- A high concentration of St. John's wort damages reproductive cells and has an effect on fertility.
- Common: Headache, nausea, abdominal discomfort, constipation, dizziness, confusion, fatigue, dry mouth, sleep disturbances, and sedation.
- Infrequent: Photosensitivity or photodermatitis, elevated liver function tests, acute neuropathy, increased PT.

DRUG INTERACTIONS

- MAOI-concomitant use with MAOIs such as tranylcypromine, phenelzine may lead to increased effects and possible toxicity (hypertensive crisis).
- Prudent to avoid concomitant use with β sympathomimetics eg, ma huang or pseudoephedrine.
- Tannic acids may interfere with the absorption of iron.
- Usage with other photosensitizers such as tetracyclines, sulfonamides, thiazides, quinolones, piroxicam, and others should be avoided
- **Cytochrome3A4**: St. John's wort has been shown to induce cytochrome isoenzyme 3A4, therefore affecting metabolism of certain medications and reducing serum concentrations. Drugs metabolized by 3A4 include:
- **Theophylline**: Blood levels of theophylline may be significantly reduced resulting in decreased efficacy.
- **HIV protease inhibitors:** Blood levels of indinavir, nelfinavir, ritonavir, and saquinavir can be significantly reduced, resulting in increased HIV viral load and development of viral resistance. Indinavir: decreases the concentration of the protease inhibitor by inducing the P450 system.
- **HIV non-nucleoside reverse transcriptase inhibitors**: Blood levels of efavirenz and nevirapine can be significantly reduced, resulting in increased HIV viral load.
- **Cyclosporin/ Tacrolimus**: Blood levels of cyclosporin or tacrolimus can be significantly reduced, resulting in decreased efficacy. Levels of cyclosporine have decreased with St. John's wort administration. St. John's wort induces cytochrome P450 enzyme system, the major pathway of cyclosporine metabolism.
- **Diltiazem / Nifedipine**: Blood levels of diltiazem or nifedipine can be reduced, resulting in decreased efficacy.
- **Irinotecan:** Due to changes in hepatic metabolism caused by St. John's wort, levels of irinotecan metabolite SN-38 may be lowered by as much as 40% for up to 3 weeks following discontinuation of St. John's wort.
- **Warfarin:** May increase or decrease activity when administered concomitantly. INR should be monitored routinely. S-isomer may have increased metabolism due to Cyp 3A4 induction. S-isomer may have decreased metabolism due to Cyp 1A2 inhibition.
- **Digoxin:** Prolonged concurrent administration may result in decreased absorption of digoxin with lowered plasma concentrations. St. John's wort decreases the effect of digoxin and [may make] a patient a non-responder whereas increased toxicity may be anticipated after withdrawal of the drug.
- **Triptans:** Increased serotonergic effect and possible serotonin syndrome when combined with sumatriptan, naratriptan, rizatriptan, or zolmitriptan.
- **SSRIs**: Increased serotonergic effect and possible serotonin syndrome when combined with citalopram, fluoxetine, fluvoxamine, paroxetine, or sertraline.
- St. John's wort taken along with SSRI such as fluoxetine, paroxetine, sertraline, fluvoxamine or citalopram leads to an increased effect and possible toxicity "serotonin syndrome" eg, sweating, tremor, flushing, confusion, and agitation.
- **Tricyclic antidepressants:** Increased serotonergic effect and possible serotonin syndrome when combined with nefazodone, amitriptyline, or imipramine. Possible reduction in efficacy of antidepressants due to changes in metabolism.
- **Oral contraceptives:** May reduce blood levels resulting in decreased efficacy (ie, breakthrough bleeding or pregnancy).
- **Alcohol:** May result in increased sedation.
- **Anesthetics:** Case report of cardiovascular collapse (hypotension without anaphylactic symptoms) shortly after induction of general anesthesia with fentanyl, propofol, d-tubocurarine, and succinylcholine followed by nitrous oxide, oxygen and isoflurane.
- **Chemotherapy:** Due to changes in hepatic metabolism caused by St. John's wort, chemotherapy levels may be altered, resulting in increased toxicity or decreased efficacy. Caution should be exercised when administering concomitantly with chemotherapy (ie, cyclophosphamide, paclitaxel, etoposide, irinotecan).
- **Tamoxifen:** Due to changes in hepatic metabolism caused by St. John's wort, levels of tamoxifen may be lowered, resulting in reduced efficacy.
- **Sympathomimetics**: Concomitant administration may produce increased serotonergic activity and possible serotonin syndrome.
- Hypericin causes a reduction in barbiturates-induced sleeping times.

DAILY DOSE

- In general, 200-1000 micrograms of hypericin is recommended for treatment of depression for 4-6 weeks.
- 300 mg of standardized extract should be administered three times daily.
- Dried herb-2 to 4 grams 3 times daily.
- Tea-single dose of 2-3 gms dried herb.
- Liquid extract-1:1 in 25 % ethanol - 2-4 ml, 3 times daily.
- Tincture-2-4 ml, 3 times daily.

## Appendix 2

### Critical Facts: Ginkgo *(ginkgo biloba)*

INTRODUCTION

- Also known as fossil tree, maidenhair tree, kew tree, bai guo ye, yinhsing
- Ginkgo biloba extract (GBE) is used to treat cerebral circulation, dementia, peripheral vascular disorders, sexual dysfunction resulting from selective serotonin reuptake inhibitors (SSRIs), hearing loss, and more.

PURPORTED USES

- Anxiety, asthma, bronchitis, cardiovascular disease, circulatory disorders, hearing loss, memory loss, Raynaud's disease, sexual dysfunction, stress, tinnitus.

WARNINGS

- Ginkgo biloba extracts should not contain ginkgolic acid.
- Discontinue ginko biloba at least 36 hours before surgery.

PRECAUTIONS AND ADVERSE REACTIONS

- Common: Headache, dizziness, GI upset, flatulence, diarrhea, contact dermatitis, and palpitations.
- Fertility: Ginkgo has adverse effects on oocytes.
- Case reports: Seizures have occurred in patients predisposed to seizures or on medications that lower the seizure threshold (eg, prochlorperazine, chlorpromazine, perphenazine, etc.). Spontaneous bleeding, including hematomas and hyphema, has been noted in the literature.

DRUG INTERACTIONS

- **Monoamine oxidase inhibitors (MAOIs):** Ginkgo may potentiate the effects of MAOIs.
- **Anticoagulants / Antiplatelets:** Ginkgo may induce spontaneous bleeding possibly associated with reduced platelet aggregation resulting from inhibition of platelet activating factor by ginkgolide components.
- **Antipsychotics / Prochlorperazine:** Ginkgo may cause seizures when combined with medications that lower the seizure threshold.
- **Insulin:** Ginkgo can alter insulin secretion and effect blood glucose levels.
- **Cytochrome P450:** Preliminary evidence that ginkgo can affect the cytochrome enzymes 1A2, 2D6, and 3A4, however controversial data exist whether it induces or inhibits the individual enzymes.
- **Trazodone:** Ginkgo extract was associated with coma in a woman with Alzheimer's disease who was also taking trazodone.

CONTRAINDICATIONS

- Patients sensitive to ginkgo.
- Patients with known risk factors for intracranial hemorrhage (hypertension, diabetes amyloid senile plaques) should avoid gingko.

## Appendix 3

### Critical Facts: Ginseng

A) GINSENG*

DAILY DOSE

- Average daily dose is 1-2 gms root. Infusion may be taken 3 to 4 times a day over 3 to 4 weeks.

INDICATIONS AND PURPORTED USES

- Lack of stamina-fatigue and debility, unproven uses-loss of appetite, cachexia, impotence and sterility, neuralgia, and insomnia.
- Chinese medicine-hemoptysis, gastric disturbances and vomiting.
- Homeopathic-rheumatism and debility.

PRECAUTIONS AND ADVERSE REACTIONS

- General-[to be taken with] caution [by] patients with cardiovascular diseases or diabetes. Hypertension resulting from ginseng abuse syndrome is associated with prolonged high dose ginseng with concomitant use of caffeine. General adverse effects include insomnia, epistaxis, headache, nervousness, and vomiting.
- Mastalgia with diffuse breast nodularity.
- Vaginal bleeding-oral ginseng and ginseng face cream have been associated with post menopausal vaginal bleeding.
- Pregnancy and lactation-maternal use has been associated with neonatal androgenization and it is therefore not recommended for use during pregnancy.
- **Overdoses**-massive overdoses bring about ginseng abuse syndrome characterized by hypertension, insomnia, hypertonia, and edema.

DRUG INTERACTIONS

- Diabetes drugs/ insulin-ginseng has been shown to have hypoglycemic effects.
- Warfarin/ NSAIDS?Antiplatelet agents-ginseng has an anti-platelet effect and [is] to be avoided along with antiplatelet agents/ NSAIDS.
- Phenelzine-headache, tremors, and mania.
- Loop diuretics-germanium (present in most ginseng products) causes loop resistance. Germanium causes nephrotoxicity in the nephron segment where loop diuretics work.

B) ASIAN GINSENG (p*anax ginseng*)*

INTRODUCTION

- Also known as Chinese ginseng, panax, ren shen, jintsam, ninjin, Asiatic ginseng, Japanese ginseng, Oriental ginseng, Korean red ginseng.
- Patients take this supplement to improve athletic performance, strength and stamina, and as an immunostimulant for diabetes, cancer, HIV/AIDS, and a variety of other conditions. It is also widely used as a "Yang" tonic in Chinese herbal formulas.

PURPORTED USES

- Angina, diabetes, health maintenance, HIV and AIDS, immunostimulation, improve clotting, pain, sexual dysfunction, strength and stamina.

WARNINGS

- Discontinue ginseng at least one week before surgery.

DRUG INTERACTIONS

- Monoamine oxidase inhibitors (MAOIs): Panax ginseng may cause manic-like symptoms when combined with MAOIs.
- Insulin and sulfonylureas: Panax ginseng may increase the hypoglycemic effect of insulin and sulfonylureas.
- Anticoagulants: Panax ginseng may antagonize the effects of anticoagulants.

CONTRAINDICATIONS

- Panax ginseng may have estrogenic activity, but data are inconsistent. Patients with hormone-sensitive disease should not consume panax ginseng.

ADVERSE REACTIONS

[Usually well tolerated.]

- Reported: Dry mouth, tachycardia, nausea, vomiting, diarrhea, insomnia, and nervousness.

**C) AMERICAN GINSENG**

INTRODUCTION

- Patients take this supplement to improve athletic performance, strength, and stamina, and to treat diabetes and cancer. In Chinese herbal formulas, American ginseng is frequently used to nourish "Yin."

PURPORTED USES

- Cancer prevention, cancer treatment, diabetes, health maintenance, immunostimulation, strength and stamina.

ADVERSE REACTIONS

- No significant reactions reported.

DRUG INTERACTIONS

- Monoamine oxidase inhibitors (MAOIs): American ginseng may cause manic-like symptoms when combined with MAOIs.
- Insulin and sulfonylureas: American ginseng may increase the hypoglycemic effect of insulin and sulfonylureas.
- Anticoagulants: Theoretically, American ginseng may antagonize the effects of anticoagulants.

D) SIBERIAN GINSENG (*eleutherococcus senticosus, acanthopanax senticosus*)

PURPORTED USES

- Chemotherapy side effects, health maintenance, immunostimulation, strength and stamina.

WARNINGS

- Case reports in the literature suggest possible contamination with incorrect botanical.
- Analysis of product suggests that labeled concentration differs from listed or assumed contents.
- Products should be tested and standardized to ensure purity and accuracy of content.

CONTRAINDICATIONS

- Patients with hypertension should not consume ginseng.

ADVERSE REACTIONS

- Reported: Insomnia, drowsiness, nervousness, tachycardia, headache, hypoglycemia.

DRUG INTERACTIONS

- Insulin / hypoglycemics: Theoretical additive hypoglycemic effect.
- Caffeine: May have additive effect leading to insomnia or nervousness.
- Hexobarbital: Eleuthero inhibits metabolism possibly by inhibition of cytochrome p450 2C19.
- Digoxin: Elevate[s] serum digoxin levels.

*We evaluated Web sites with content on ginseng using the general ginseng critical facts and Web sites with content on the specific types of ginseng (Asian, American, and Siberian) with the critical facts on the specific types of ginseng.

## Abbreviations

BI: Bias index
CAM: Complementary and alternative medicine
MAOI: Monoamine oxidase inhibitor
NSAIDS: Non-steroidal aniti-imflammatories
PABAK: Prevalence-adjusted bias-adjusted kappa
PI: Prevalence index
SPSS: Statistical Package for the Social Sciences
SSRI: Selective serotonin reuptake inhibitor
